# Molecular connections between nuclear and ciliary import processes

**DOI:** 10.1186/2046-2530-2-11

**Published:** 2013-08-28

**Authors:** H Lynn Kee, Kristen J Verhey

**Affiliations:** 1 Department of Cell and Developmental Biology, University of Michigan Medical School, Ann Arbor, MI 48109, USA

**Keywords:** Cilia, Ciliary pore complex, Flagella, Nuclear import, Nuclear pore complex, Nucleoporin, Ran, Size exclusion

## Abstract

As an organelle, the cilium contains a unique complement of protein and lipid. Recent work has begun to shed light on the mechanisms that regulate entry of ciliary proteins into the compartment. Here, we focus on the mechanisms that regulate ciliary entry of cytosolic molecules. Studies have revealed a size exclusion mechanism for ciliary entry that is similar to the barrier to nuclear entry. Active import into the ciliary compartment involves nuclear trafficking components including importins, a Ran-guanosine triphosphate gradient, and nucleoporins. Together, this work indicates that nuclei and cilia share molecular, structural and mechanistic components that regulate import into the compartments.

## Review

### Introduction

Eukaryotic cells have evolved to maintain specialized functions and morphologies by compartmentalizing cellular activities within topologically distinct organelles such as the nucleus, mitochondrion and endoplasmic reticulum. Recent work has suggested that the cilium is also a specialized organelle. Cilia and flagella are microtubule-based organelles that protrude from the cell surface and function in cellular motility and extracellular sensing. For example, motile cilia (or flagella) beat to move mucus up the respiratory tract, establish left-right asymmetry in the embryonic node, and propel sperm. Non-motile cilia, also called primary or sensory cilia, were once believed to be vestigial organelles without complex function. They are now known to act as cellular ‘signaling antennas’ responsible for a variety of functions including olfaction in olfactory neurons, photoreception in photoreceptor cells, mechanosensing of fluid flow in kidney epithelial cells, and responding to extracellular signals like Hedgehog, Wnt and platelet-derived growth factor ligands (reviewed in [1,2]). The modern view of primary cilia as sensory antennae has been driven by recent findings that defects in ciliary formation, function and/or signaling underlie a group of phenotypically diverse disorders now known as ciliopathies [3,4].

An important characteristic of the cilium or flagellum is that the organelle protrudes from the cell surface such that the ciliary membrane is continuous with the plasma membrane and the intraciliary space is exposed to the cytosolic space. This raises the important question of how ciliary components are targeted to and/or retained in the organelle. For example, structural components such as the outer dynein arm and radial spoke complexes of motile cilia are assembled in the cytosol and trafficked specifically to the cilium [5,6]. In addition, the enrichment of many membrane and soluble signaling factors in the ciliary compartment is required for proper motile and sensory function. For example, in the Hedgehog pathway, trafficking of soluble Gli transcription factors through the ciliary compartment is required for proper Gli proteolysis and subsequent transcriptional output [7,8].

**Figure 1:**
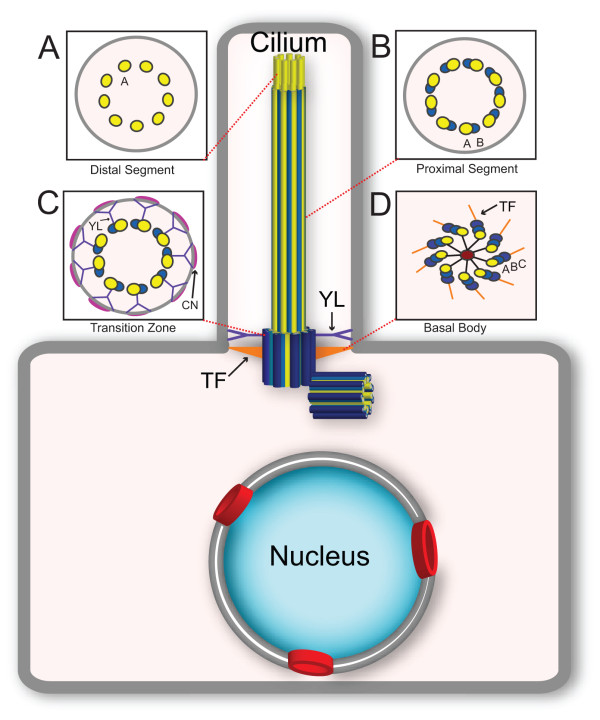
**General structure of the cilium.** The structural core of the cilium consists of a microtubule-based axoneme, which protrudes from the mother centriole in the basal body. Insets depict cross-sections of the microtubule structure along the distal to proximal ciliary axis. **(A)** Nine single microtubules of the distal segment. **(B)** Nine doublet microtubules of the core axoneme. **(C)** The transition zone contains Y-link structures (YL, purple) that link the axoneme to the membrane as well as membrane protrusions termed the ciliary necklace (CN, pink). **(D)** The basal body consists of nine triplet microtubules of the mother centriole and associated transition fibers (TF, orange).

Entry into the ciliary compartment takes place at a region at the base of the cilium termed the transition zone, where the basal body transitions into the axoneme (Figure 1). Structurally, the transition zone is characterized by transition fibers and Y-link structures that link the basal body/axoneme to the membrane and by membrane protrusions termed the ciliary necklace (reviewed by [9,10]; Figure 1). It was hypothesized that the transition fibers might be components of a flagellar/ciliary pore complex (CPC) that controls the entry of ciliary proteins in a sieve-like manner, analogous to the way that nuclear pore complexes (NPCs) control entry of cytosolic components into the nucleus [11,12]. What are the molecules that comprise these structures and what are their roles in ciliary gating? Recent work has identified several classes of proteins that localize to the transition zone and play a role in gating: ciliopathy gene products (for example, nephronophthisis (NPHP) and Meckel-Gruber Syndrome (MKS) proteins), nucleoporins, and septins (reviewed by [9,10]).

Two pathways for ciliary trafficking need to be considered - entry and exit of membrane proteins, and entry and exit of cytosolic proteins. Several lines of evidence support the idea that ciliopathy gene products and septins play important roles in regulating the entry and exit of membrane proteins [13-17]. In this review, we will focus on the trafficking of cytosolic proteins into the ciliary compartment.

### Is there a barrier for entry of soluble proteins into the ciliary compartment?

As the intraciliary space appears to be continuous with that of the cytosolic space, whether entry of cytosolic components into the ciliary compartment is restricted is an important question. Using soluble GFP (approximately 27 kDa, 4.2 nm × 2.4 nm barrel) as a model protein in *Xenopus* photoreceptor cells, Calvert *et al*. showed that the connecting cilium (the transition zone equivalent) provides only a modest barrier to diffusion between the inner and outer segments [18]. Further work showed that tandem GFP proteins, 2xGFP (approximately 54 kDa) and 3xGFP (approximately 81 kDa), freely entered the outer segment compartment, albeit to a lesser extent than single GFP [19]. This work concluded that no diffusion barrier exists to regulate the entry of cytosolic proteins into the ciliary compartment, at least for proteins of up to approximately 80 kDa. Rather, size-restricted flux into photoreceptor outer segments was postulated to be due to steric volume exclusion within this compartment [20]. In this model, the membranous discs and high protein concentration in the outer segment reduce the aqueous volume available to soluble molecules such that larger molecules will be less abundant in this environment than smaller proteins.

**Figure 2:**
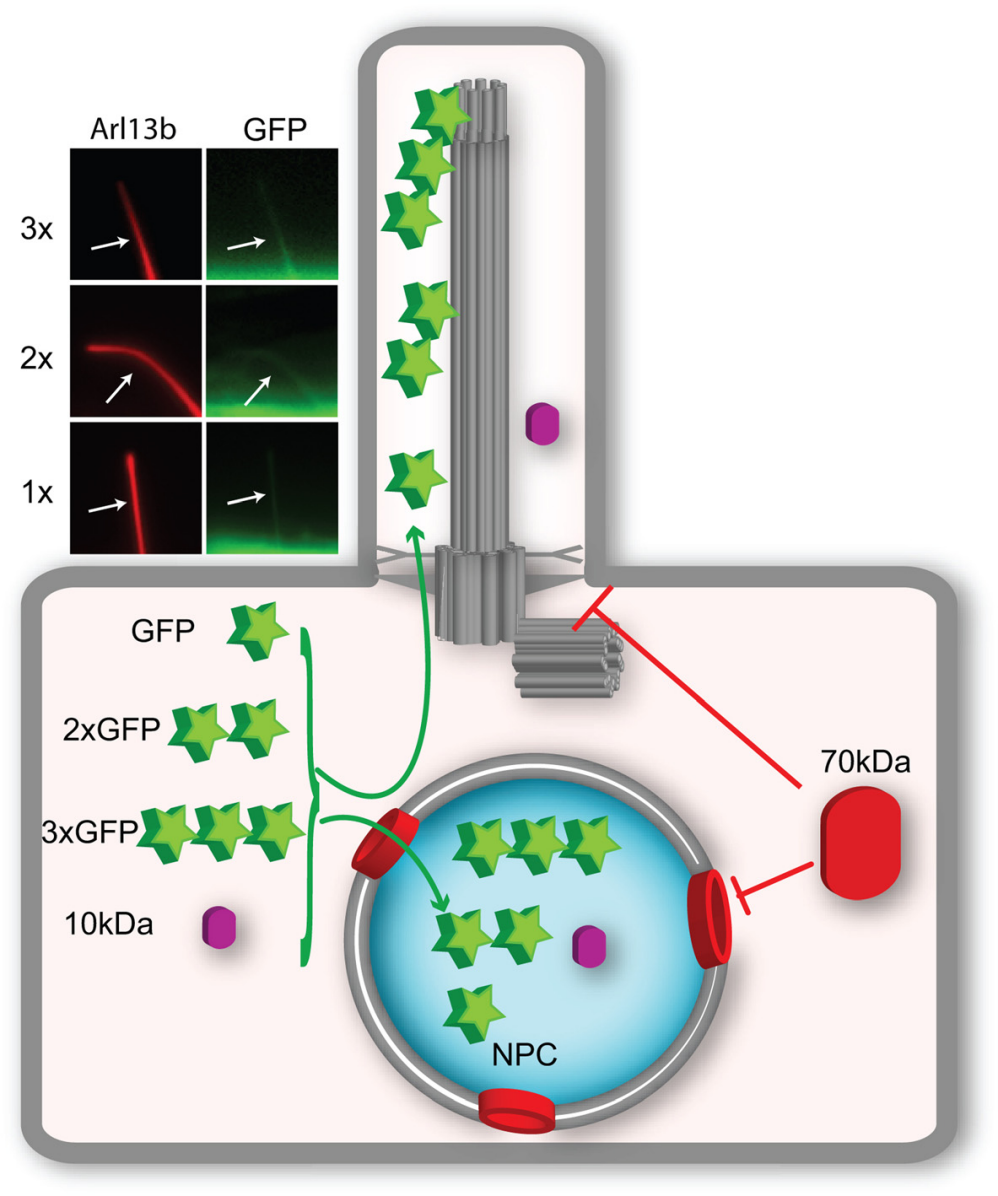
**Model of the size-dependent diffusion barrier at the base of the cilium.** The base of the cilium contains a size-dependent barrier to entry of soluble proteins. Molecules that are 10 kDa (purple) can enter both the cilium and nucleus but 70 kDa (red) molecules are restricted from both compartments. Insets shows fluorescence micrographs of the cilia of NIH3T3 cells co-expressing monomeric GFP (1x) or tandem (2x or 3x) GFPs together with Arl13b (red) to mark the ciliary compartment. Despite the difference in molecular weight, monomeric and tandem fluorescent protein constructs can enter the ciliary compartment, presumably due to their similar diameters. GFP, green fluorescent protein; NPC, nuclear pore complexes.

To test whether a diffusion barrier exists for entry of cytosolic proteins into primary cilia in mammalian cells, we utilized a microinjection approach based on classic experiments that demonstrated a size-exclusion barrier for entry into the nuclear compartment. Fluorescent dextrans of different molecular weights were microinjected into the cytosol of hTERT-RPE cells. Small (3 and 10 kDa) dextrans were observed to enter both nuclear and ciliary compartments whereas larger (40 and 70 kDa) dextrans were excluded from both compartments [21] (Figure 2). Further work examined the ability of fluorescently labeled soluble proteins to enter the ciliary compartment and a similar size-based restriction against passive diffusion into the cilium was observed. Small proteins (approximately 14 to 41 kDa) entered both the nuclear and ciliary compartments whereas a larger protein (approximately 67 kDa) was excluded from both compartments [21]. Thus, in contrast to the results of Najafi *et al*. [19], these studies indicated that a barrier to entry exists for entry of molecules larger than approximately 50 kDa into the ciliary compartment.

One possible explanation for the differences between the work of Najafi *et al*. [19] and Kee *et al*. [21] is the transport substrate, in that the former study used proteins linked as beads on a string and the later study used globular proteins of different sizes. To directly compare entry into the primary cilium to that of photoreceptors, we created fusion proteins containing tandem fluorescent proteins (FPs). Like single GFP, proteins consisting of two FPs (approximately 54 kDa) or three FPs (approximately 81 kDa) were able to enter into primary ciliary (Figure 2). Although fusing FPs in tandem increases the molecular weight and the length of the molecule in a linear fashion, the width of the single and tandem FPs are the same and they are therefore able to cross the diffusion barrier and enter the outer segment of photoreceptor cells [19] and primary cilia of hTERT-RPE cells (Figure 2). Collectively, this work indicates that a ciliary barrier restricts the free entry of soluble proteins into the compartment and that a variety of features, including molecular weight and the overall structural conformation of a transport substrate, impact a molecule’s ability to cross this barrier.

A recent study approached the issue of access of soluble proteins to the ciliary compartment by using a high-affinity interaction induced by the drug rapamycin to trap soluble proteins that diffuse into primary cilia [22]. This technique allowed the authors to specifically measure the kinetics of ciliary accumulation of proteins of various sizes. The authors found that steric volume exclusion is not likely to be a defining feature of the barrier in primary cilia. Rather, the ciliary barrier was found to behave like a molecular sieve in that the entry of proteins into primary cilia was restricted in a size-dependent manner. The major discrepancy with the work of Kee *et al*. [21] appears to be in the size for restricted entry; Lin *et al*. [22] found that large multimeric complexes up to 8 nm in radius and 650 kDa in size could become trapped in the cilium.

Two parameters must be kept in mind when evaluating the differences between these studies. The first is experimental. Each of the experimental setups (microinjection and dimerization-induced trapping) has its drawbacks. Whereas the trapping of FPs in the ciliary compartment enables better visualization of the ciliary proteins over the cytosolic pool (a major limitation in the microinjection system), the use of a membrane protein as an anchor for the ‘trap’ may cause aberrant entry of large cytosolic proteins into the ciliary compartment. Clearly, more work is needed to define the physical properties of the ciliary barrier. The second parameter that must be considered is that factors in addition to molecular weight are likely to influence protein mobility and movement through the pore.

Collectively, these experiments demonstrate that entry of soluble proteins into the ciliary compartment is restricted by a size-based exclusion mechanism. This is reminiscent of entry into the nucleus, which has mechanisms in place to prevent entry of cytosolic molecules. Protein gateways, the NPCs, span the nuclear envelope and create pores that function to control the exchange of molecules between the cytoplasm and nucleoplasm. The NPC forms a permeability barrier and allows the diffusional entry of small molecules (<40 kDa) but hinders the passage of larger molecules, thus maintaining the nucleus as a privileged domain with unique composition [23-25]. This protects the eukaryotic cell’s genetic material and transcriptional machinery, and ensures proper functioning of nuclear activities.

### Nucleoporins constitute a ciliary pore complex at the base of the cilium

What are the molecular components of the diffusion barrier at the base of cilia? Nucleoporin proteins make up the NPCs that are embedded in the nuclear envelope and regulate entry into this compartment [26-28]. Recent work has shown that endogenous and expressed nucleoporins also localize to the base of primary and motile cilia in mammalian cells [21] to form a CPC. Furthermore, nucleoporin function is required for the gated entry of the cytosolic kinesin-2 motor KIF17 into the ciliary compartment [21]. Although further work is needed to verify and extend these results in other ciliated cells, this work demonstrates that the nuclear and ciliary barriers share molecular components that regulate organelle composition. These results raise many interesting questions about the molecular, structural and evolutionary relationships between the NPC and CPC.

**Figure 3:**
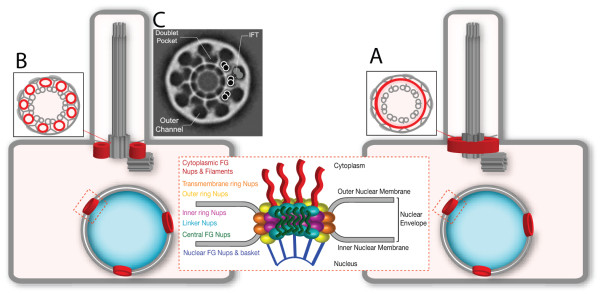
**Nucleoporins in cilia and nuclei.** Nuclear pore complexes (red donuts in nuclear envelope) contain nucleoporin proteins that assemble into subcomplexes (center). Some nucleoporin subcomplexes also localize to the transition zone where they are postulated to form a ciliary pore complex (red donuts at ciliary base). Two possible structural configurations of the nucleoporins at the base of the cilium are presented. **(A)** Model in which nucleoporins assemble into one large pore at the base of the cilium with the axoneme protruding through the middle of the pore. **(B)** Model in which nucleoporins assemble into nine pores at the base of the cilium with each pore positioned between the Y-links. **(C)** Electron cryotomography analysis of isolated basal body structures from the protist *Tetrahymena pyriformis* indicates nine pore structures adjacent to the microtubule axonemes. FG, phenylalanine-glycine. Reprinted from Ounjai *et al*. [35], with permission from Elsevier.

Each NPC is composed of multiple copies of approximately 30 different nucleoporins that assemble into distinct subcomplexes with specific roles within the NPC (Figure 3) [29]. Interestingly, the NPC and CPC may not be identical in molecular composition as not all NPC subcomplexes were found to localize to the base of primary cilia in cultured cells [21]. For example, nucleoporins that contain largely unstructured repeats form the actual barrier of the NPC and were also found to localize to the ciliary base. By contrast, nucleoporins of the nuclear basket subcomplex form a platform for nuclear-specific activities but were not found at the base of primary cilia in cultured cells. Likewise, the transmembrane nucleoporins that anchor the NPC in the nuclear membrane did not localize to the ciliary base in cultured cells, suggesting that alternative mechanisms may recruit and anchor nucleoporins in the plasma membrane at the base of the cilium. If this is true, then identification of the ciliary transmembrane anchor proteins is an important goal. One potential anchor is the NPHP/MKS complex of proteins that localizes to the cilia base and has been implicated in ciliary gating (reviewed in [10]). Seven proteins in the NPHP/MKS complex have predicted transmembrane domains and their localization to the transition zone would allow them to anchor the CPC at this locale. Another important goal is to fully determine the nucleoporin composition of the CPC across cell types and tissues as there may be heterogeneity in CPC composition and function like there is for the NPC [30].

Another important question concerning the relationship between the NPC and the CPC concerns the overall structure of the CPC. Each NPC has typically an eight-fold rotational symmetry [31,32], although pores with nine- or ten-fold symmetry have been noted [33,34]. By contrast, the cilium is characterized by nine-fold symmetry due to the core microtubule doublets of the axoneme (Figure 1). It is not clear whether the difference between the eight-fold symmetry of the NPC and the nine-fold symmetry of the cilium is important, as we do not have any information about how the nucleoporin subunits are arranged at the base of the cilium to form an actual pore. One possibility is that there is one large pore at the base of the cilium with the axoneme protruding through the middle of the pore (Figure 3A). Such a pore would presumably have a nine-fold symmetry based on that of the axoneme. An alternative possibility is that there are nine pores positioned between the Y-links at the base of the cilium (Figure 3B)*.* In this scenario, each CPC would retain the characteristic eight-fold symmetry of the NPC. In support of this possibility, recent electron cryotomography analysis of isolated basal body structures from the protist *Tetrahymena pyriformis* demonstrated the presence of an electron-dense ‘terminal plate’ structure that spans the ciliary base and contains nine pore structures, one adjacent to each microtubule doublet of the axoneme (Figure 3C) [35]. Are these *Tetrahymena* CPCs of the terminal plate the same barriers as the nucleoporin-containing CPCs found in mammalian primary and motile cilia? One striking finding in support of this is that the CPCs in the *Tetrahymena* terminal plate have a diameter of approximately 53 nm, similar to the pore diameter of mammalian NPCs [36]. In addition, proteomic analysis of the isolated *Tetrahymena* basal bodies identified proteins involved in nuclear transport including Ran and the transmembrane nucleoporin NDC-1 [35]. Further proteomic and structural analysis will reveal the exact molecular composition of the CPC and its organization at the ciliary base.

The shared gating mechanism of nuclei and cilia has evolutionary implications as well. Cilia are found in a wide range of eukaryotic taxa and were already present in the last eukaryotic common ancestor [37]. Unlike nuclei, cilia were then independently lost from multiple eukaryotic lineages (for example, fungi, amoebae and some plants) [38,39]. Recent work has uncovered structural and sequence similarities between outer ring nucleoporins, intraflagellar transport (IFT) proteins, and vesicle coat proteins (COPs and clathrins) [40-44]. These findings have led to the hypothesis that a ‘protocoatamer’ gave rise to membrane-coating components during eukaryotic evolution [45,46]. It thus appears that the evolutionary appearance of both nuclei and cilia involved the adaptation of an ancestral protocoatamer component into both gating (NPC and CPC) and trafficking (IFT, coatamer) components.

### Active transport of soluble proteins into the ciliary compartment

Gated entry into the nuclear and ciliary compartments has shared mechanisms beyond the size-exclusion barrier and nucleoporin-containing pore complexes. Entry of proteins above the size barrier into the nuclear compartment requires an active transport mechanism involving cytosolic recognition of nuclear localization sequences (NLS) by transport receptors called importins (or karyopherins), shuttling across the NPC, and release of NLS-containing proteins in the nuclear compartment by the small G protein Ran. Interestingly, entry of cytosolic proteins into the ciliary compartment has also been shown to utilize an NLS-like signal, importins and Ran.

Two classes of NLS have been described. First, the classical NLS consists of one or two stretches of basic residues that bind directly to an importin-α adaptor protein and thereby indirectly to importin-β1 in order to traverse the NPC. The best-studied NLSs of this class are the monopartite sequence of the SV40 large T antigen and the bipartite sequence of nucleophosmin [47]. Second, nonclassical NLSs have diverse amino acid sequences that bind directly and specifically to other members of the importin-β family. Best-studied in this class is the M9 sequence from the heterogeneous nuclear ribonucleoprotein A1 protein, which binds directly to importin-β2 (transportin-1) [48].

**Figure 4:**
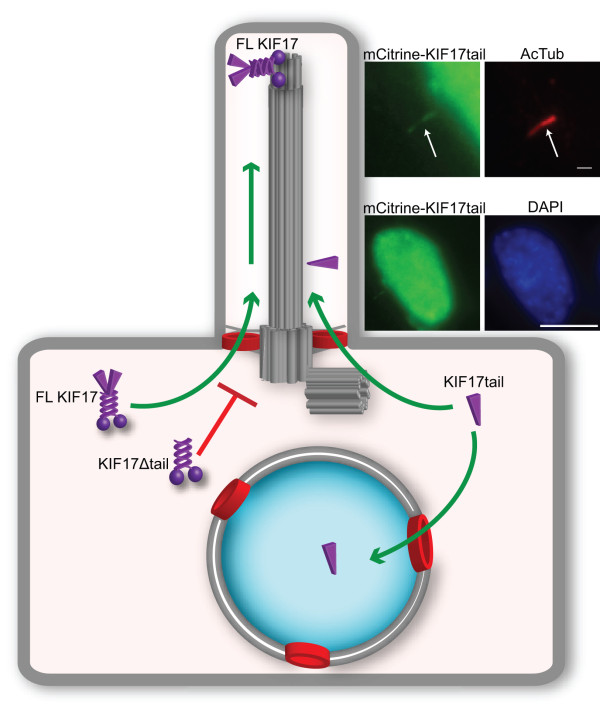
**A ciliary localization sequence regulates ciliary localization of the kinesin-2 motor KIF17.** Full-length KIF17 can enter the ciliary compartment whereas a truncation that removes the tail domain (KIF17Δtail) abolishes ciliary localization. When expressed as a fragment, the KIF17 tail domain localizes to both the ciliary (inset top, cilia immunostained with acetylated tubulin antibody in red) and nuclear (inset bottom, nucleus stained with DAPI in blue) compartments, due to the presence of a sequence that can act as a ciliary localization sequence and nuclear localization sequence.

Ciliary targeting via NLSs was first described for an IFT component, the kinesin-2 motor KIF17. IFT is the bidirectional transport of ciliary components along axonemal microtubules by kinesin and dynein motors. The motors and their IFT cargoes are large macromolecular complexes, well above the size exclusion barrier for entry into the ciliary compartment. Dishinger *et al.* found that full-length kinesin-2 KIF17 accumulates at the tip of the cilium in various cell lines but that removal of the C-terminal tail domain blocks ciliary localization [49]. Further work showed that the C-terminal tail domain of KIF17, which localizes to both the nuclear and ciliary compartments (Figure 4), contains a classical NLS that binds to importin-β2 [49]. This sequence serves as a NLS for entry of the tail fragment into the nuclear compartment and as a ciliary localization sequence for entry of the full-length molecule into the ciliary compartment (Figure 4). This result has two important implications. First, the same signal can serve as an NLS or ciliary localization sequence depending on protein context. Second, additional sequences in KIF17 are required for ciliary targeting of the full-length motor and perhaps its associated cargoes.

Further work demonstrated that an NLS and importin-β2 are required for ciliary entry of retinitis pigmentosa 2 (RP2), a lipid-anchored peripheral membrane protein [50]. In this case, both classical and nonclassical NLS sequences were identified in the retinitis pigmentosa 2 primary sequence and mutational analysis determined that the nonclassical sequence is critical for mediating ciliary entry of retinitis pigmentosa 2 [50]. That a nonclassical NLS binds to importin-β2 and mediates transport across the CPC parallels what has been observed for nuclear import. The fact that KIF17 appears to use a classical NLS to interact with importin-β2 and traverse the CPC is puzzling. Further mutational analysis of the KIF17 NLS is required to define the sequence parameters that mediate the interaction with importin-β2 and ciliary entry.

Importin-β1 has been shown to bind to the ciliary transmembrane proteins Crumbs [51] but whether this interaction regulates ciliary entry is unknown. Expression of dominant negative importin-β1 or knockdown of the endogenous protein resulted in defects in ciliogenesis [51], suggesting that importins and their cargoes play important roles in ciliary processes in addition to regulating ciliary entry.

### A Ran gradient for directional transport

**Figure 5:**
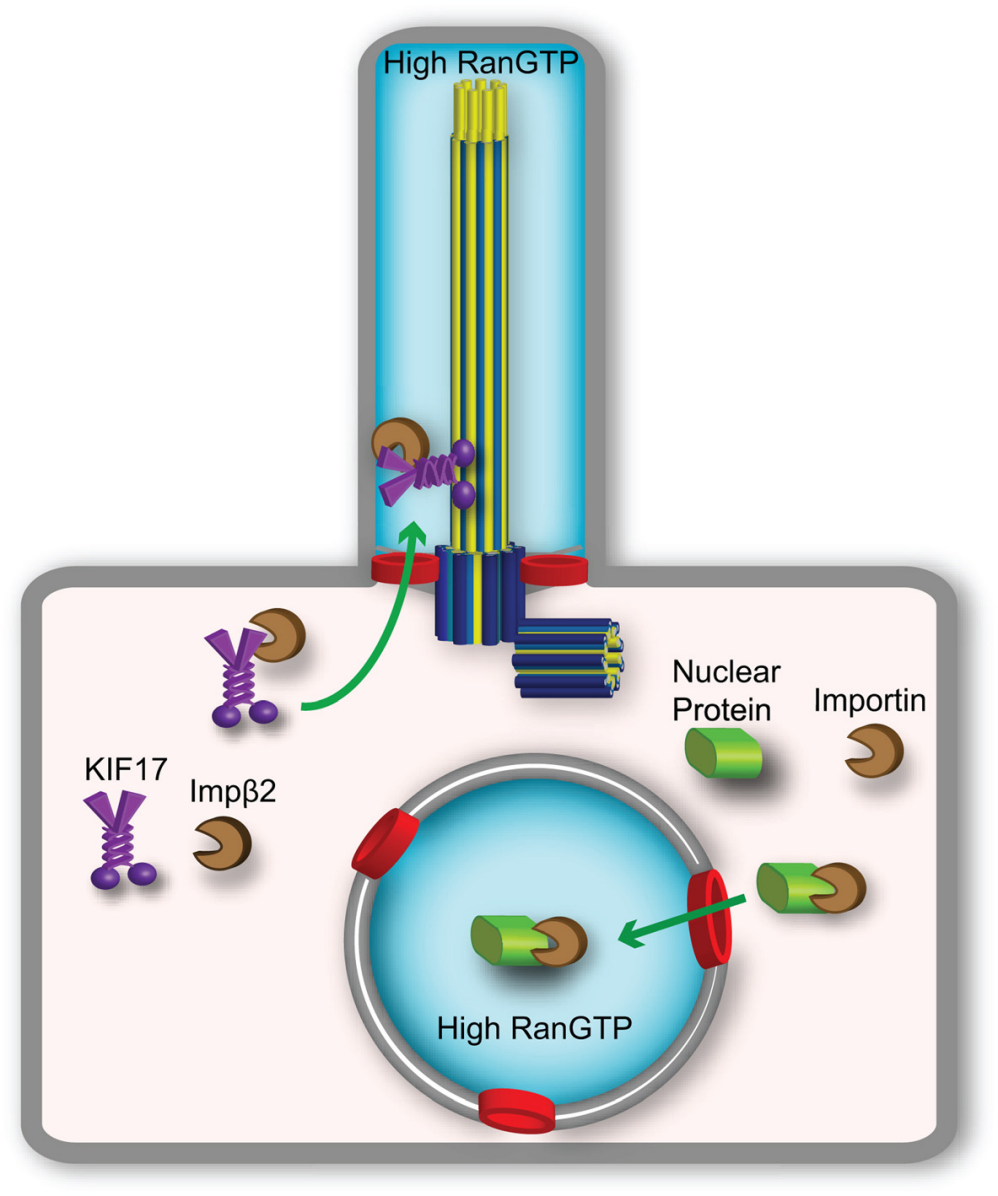
**A RanGTP gradient regulates ciliary and nuclear trafficking.** For ciliary trafficking, the ciliary localization sequence of KIF17 interacts with Importin-β2 for trafficking through the ciliary pore complex into the ciliary compartment where the high Ran-GTP concentration (blue shading) dissociates the complex. For nuclear trafficking, the nuclear localization sequence of a nuclear protein interacts with an importin receptor for trafficking across the nuclear pore complex into the nuclear compartment where the high RanGTP concentration (blue shading) dissociates the complex. RanGTP, Ran-guanosine triphosphate.

The directionality of nuclear-cytoplasmic trafficking is regulated by the small G protein Ran. High levels of Ran-guanosine diphosphate (GDP) in the cytoplasm promote the association of importins and their NLS-containing cargoes whereas high levels of Ran-guanosine triphosphate (GTP) in the nucleoplasm cause dissociation of importins from their cargoes (Figure 5). Several lines of evidence indicate that a RanGTP/GDP gradient also controls ciliary-cytoplasmic trafficking. RanGTP localizes to the ciliary compartment of both primary and motile cilia [49,52] (Figure 5). Disrupting the ciliary-cytoplasmic RanGTP/GDP gradient by increasing the cytosolic levels of RanGTP blocks ciliary import of KIF17 [49,52]. Furthermore, computer modeling of IFT and flagellar length control suggests that ciliary RanGTP can act as a flagellar length sensor and regulate the release of IFT particles at the flagellar base [53]. Future studies to test this model will reveal Ran’s increasing role in regulating ciliary trafficking.

An important question is how the ciliary RanGTP/GDP gradient is generated. Cytosolic RanGDP is generated, at least in part, by Ran GTPase activating protein and its cofactor RanBP1 (reviewed in [54]). Recent work suggests that RanBP1 also plays a role in regulating the ciliary RanGTP/GDP gradient as altering the levels of Ran binding protein 1 had distinct consequences for ciliogenesis [52]. Nuclear RanGTP is generated by the guanine nucleotide exchange factor (GEF) RCC1. As a chromatin-bound protein, RCC1 is localized to the nucleus. Whether RCC1 also functions as a ciliary GEF for Ran or whether a cilia-specific GEF exists is unknown. Ciliary proteomes contain both RCC1 and the related protein RCC2 as well as several proteins with tandem RCC1 repeats, including X-linked retinitis pigmentosa GTPase regulator and Secretion-regulating guanine nucleotide exchange factor [55,56]. Therefore, identifying the ciliary RanGEF is one of the next key experiments.

In addition to regulating trafficking across the ciliary-cytoplasmic barrier, recent work has shown that Ran regulates ciliogenesis in specific cell types. Ran has been localized to the centrosomes of elongating rat spermatids [57]. In cultured hTERT-RPE cells, modulating RanGTP levels through knockdown or overexpression of Ran binding protein 1 either promoted or abolished ciliogenesis, respectively [52]. As RanGTP regulates microtubule assembly during mitosis [58], it may also play a critical role in regulating microtubule assembly during axoneme formation. However, manipulating RanGTP levels in polarized MDCK cells had no effect on ciliogenesis but did significantly impair the ciliary trafficking of the kinesin-2 KIF17 motor [52]. Clearly, more work is needed to understand the role of Ran during ciliogenesis and ciliary trafficking.

## Conclusions and future directions

The work described above indicates that import into the nuclear and ciliary compartments share molecular, structural and mechanistic components. These findings raise the possibility that other regulators of nuclear-cytoplasmic trafficking may function to regulate ciliary protein localization and/or function. For example, small, ubiquitin-related modifiers (SUMOs) are approximately 100-amino-acid proteins that are covalently yet reversibly attached to substrate proteins during a variety of cellular processes including nuclear-cytoplasmic transport [59,60]. Recent work has shown that SUMOylation of the small GTPase ARL-13, the worm ortholog of Arl13B that is mutated in the ciliopathy Joubert syndrome, regulates the proper ciliary targeting of various sensory receptors and the corresponding sensory functions [61]. In addition, it seems likely that the nuclear export machinery could play a role in ciliary export processes. A recent paper suggests that phosphorylation of a potential nuclear export sequence regulates the localization of huntingtin protein to the ciliary shaft or the basal body [62].

The commonalities of nuclear and ciliary import processes raise the intriguing possibility that proteins can play functional roles in both compartments. For example, the IFT motor heterotrimeric kinesin-2 (KIF3A/KIF3B/KAP in mammals) has been found to shuttle between the nuclear and ciliary compartments in sea urchin embryos [63], although a nuclear function for kinesin-2 is not known. More established is the ciliary to nuclear shuttling of Gli transcription factors in response to extracellular Hedgehog ligand [7,8]. Furthermore, centriolar proteins such as centrins have been found to play a role in mRNA and protein transport through the NPC [64,65] and centrosomal and transition zone proteins have been found to localize to both the ciliary and nuclear compartments and have been implicated in the DNA damage response [66-69].

Both nuclear-cytoplasmic and ciliary-cytoplasmic transport events are restricted to interphase in metazoans. However, recent work has suggested that nuclear and ciliary components have important roles in the mitotic phase of the cell cycle. During mitosis, chromatin-bound RCC1 generates a spindle RanGTP gradient that activates spindle assembly factors and organizes spindle microtubules [58]. Nucleoporins such as the NUP107/160 complex relocalize to the kinetochore during prophase, where they regulate spindle assembly and establishment of microtubule/kinetochore attachments [70,71]. IFT components such as IFT88 support the formation of astral microtubules and thereby orientation of the mitotic spindle in dividing cells [72]. Other IFT proteins, including IFT27, IFT46, IFT72 and IFT139, accumulate at the cleavage furrow of dividing *Chlamydomonas* cells [73], hinting for a role of IFT proteins in cytokinesis. These and other findings that ciliary proteins have important non-ciliary functions (for example, see [74]) have broad implications in understanding the disease mechanisms for ciliopathies.

## Abbreviations

CPC: Ciliary pore complex; FP: Fluorescent protein; GDP: Guanosine diphosphate; GEF: Guanine nucleotide exchange factor; GFP: Green fluorescent protein; GTP: Guanosine triphosphate; IFT: Intraflagellar transport; MKS: Meckel-Gruber Syndrome; NLS: Nuclear localization sequence; NPC: Nuclear pore complex; NPHP: Nephronophthisis; SUMO: Small ubiquitin-related modifiers.

## Competing interests

The authors declare that they have no competing interests.

## Authors’ contributions

HLK and KJV contributed equally in writing the manuscript. Both authors read and approved the final manuscript.
